# Phenotypical and biochemical characterization of tomato plants treated with triacontanol

**DOI:** 10.1038/s41598-024-62398-0

**Published:** 2024-05-27

**Authors:** Michela Manai, Anna Fiorillo, Monica Matuozzo, Mei Li, Chiara D’Ambrosio, Loris Franco, Andrea Scaloni, Vincenzo Fogliano, Lorenzo Camoni, Mauro Marra

**Affiliations:** 1 Department of Biology, Tor Vergata University of Rome, 00133 Rome, Italy; 2 Ph.D. Program in Cellular and Molecular Biology, Department of Biology, Tor Vergata University of Rome, 00133 Rome, Italy; 3 Proteomics, Metabolomics & Mass Spectrometry Laboratory ISPAAM, National Research Council, 80055 Portici, Italy; 4 Quality and Design Group, Wageningen University & Research, 6700AA Wageningen, The Netherlands; 5 College of Food Science and Engineering, Northwest A&F University, Yangling, 712100 China; 6 IRRITEC SpA, 98070 Capo D’Orlando, Messina, Italy

**Keywords:** Plant biostimulant, *Solanum lycopersicum*, Fruit yield, Fruit nutritional properties, Abiotic stress, Tandem mass-tag proteomics, Biochemistry, Plant sciences

## Abstract

Biostimulants are heterogeneous products designed to support plant development and to improve the yield and quality of crops. Here, we focused on the effects of triacontanol, a promising biostimulant found in cuticle waxes, on tomato growth and productivity. We examined various phenological traits related to vegetative growth, flowering and fruit yield, the metabolic profile of fruits, and the response of triacontanol-treated plants to salt stress. Additionally, a proteomic analysis was conducted to clarify the molecular mechanisms underlying triacontanol action. Triacontanol application induced advanced and increased blooming without affecting plant growth. Biochemical analyses of fruits showed minimal changes in nutritional properties. The treatment also increased the germination rate of seeds by altering hormone homeostasis and reduced salt stress-induced damage. Proteomics analysis of leaves revealed that triacontanol increased the abundance of proteins related to development and abiotic stress, while down-regulating proteins involved in biotic stress resistance. The proteome of the fruits was not significantly affected by triacontanol, confirming that biostimulation did not alter the nutritional properties of fruits. Overall, our findings provide evidence of the effects of triacontanol on growth, development, and stress tolerance, shedding light on its mechanism of action and providing new insights into its potential in agricultural practices.

## Introduction

Biostimulants are natural substances from different sources applied to plants to enhance growth and development and improve fitness. Unlike fertilizers, which provide essential nutrients, biostimulants promote the biological processes of plants, enhancing nutrient uptake, improving stress tolerance, and promoting overall plant vigor^1,2^. Due to the ever-increasing demand for better yield and quality of crops as well as the need for a reduced application of chemical fertilizers, according to environment-friendly farming, the last few years have witnessed a tremendous increase in the use of biostimulants. Despite their increasing use, the effects of biostimulants on plant physiology and the activated molecular process still need a detailed characterization. This latter issue is challenging to address because, in general, biostimulants are raw blends of biological materials containing many substances with different stimulatory effects and mechanisms of action. On the other hand, there is a clear need to improve our understanding of the effects of biostimulants on a molecular basis with the aim of optimizing their use.

Triacontanol is an emerging biostimulant belonging to the botanicals category^3^ that originated in Asia, where it is widely used to enhance rice production; nowadays, its use is rapidly growing even in Western countries. This compound was isolated for the first time from alfalfa^3^; it is a long-chain primary alcohol with 30 carbon atoms, naturally occurring in waxes of the epicuticular layer of leaves of *Fabaceae* and other species^4^. Triacontanol is also described as a plant growth regulator, and different studies proved its ability to promote growth and yield of many plants, including crops, such as rice, tomato, wheat, and maize^4^. Analysis of physiological parameters demonstrated that triacontanol influences plant growth by increasing photosynthetic rate, nitrogen fixation, water, and nutrient uptake, stomata conductance, and gas exchange^4^. Physiological effects have been related at the biochemical level to a cellular increase in the content of photosynthetic pigments, soluble sugars, starch, phenols, and proteins^5^, and to stimulation of the activities of carbon and nitrogen metabolism enzymes, such as carbonic anhydrase^5,6^, nitrate reductase^6^, ribulose-1,5-bisphosphate carboxylase/oxygenase^7^, and respiratory malate dehydrogenase^8^. More recent studies documented that triacontanol improves the tolerance of plants to abiotic stresses, including salinity^9,10^, drought^11^, temperature^12^, and heavy metals^13,14^. In general, these studies demonstrated that the improvement of tolerance to stress is due primarily to the increase in the activities of antioxidant enzymes, such as superoxide dismutase, catalase, peroxidase, and those involved in the ascorbate–glutathione cycle^14^. The increase in antioxidant enzyme activity contrasts the oxidative damage, a common feature of various abiotic stresses^11^, thereby resulting in the mitigation of cell injury and the restoration of plant growth.

Minimal information is available on the molecular mechanisms underlying the action of triacontanol, including perception and signaling. Studies on rice have identified 9-L ( +) adenosine as the second messenger of the triacontanol action^15^. Applying triacontanol to leaves rapidly determined the formation of 9-L ( +) adenosine that, at nanomolar concentration, induced effects similar to those elicited by triacontanol^16^. The molecular pathways by which triacontanol determines 9-L ( +) adenosine formation and downstream events are still unknown; nevertheless, the involvement of a Ca^2+^-dependent phosphorylation signaling has been speculated^7,17^.

This work aimed to characterize the effects of triacontanol on tomato plant growth and productivity. To this purpose, different phenological traits related to both vegetative and reproductive growth were measured during biostimulation. Moreover, the effect of triacontanol on the metabolic profile of fruits and the tolerance of tomato plants to salt stress was investigated. In addition, a tandem mass tag (TMT)-based proteomic analysis of leaves and fruits was carried out to shed light on the molecular mechanism(s) underlying triacontanol action. Several differentially represented proteins (DRPs) belonging to different functional classes were identified. Their possible roles in the effect of triacontanol on growth, fruit yield, quality, and resistance to salt stress were discussed.

## Results

### Effect of triacontanol on tomato plant growth and fruit yield

**Figure 1:**
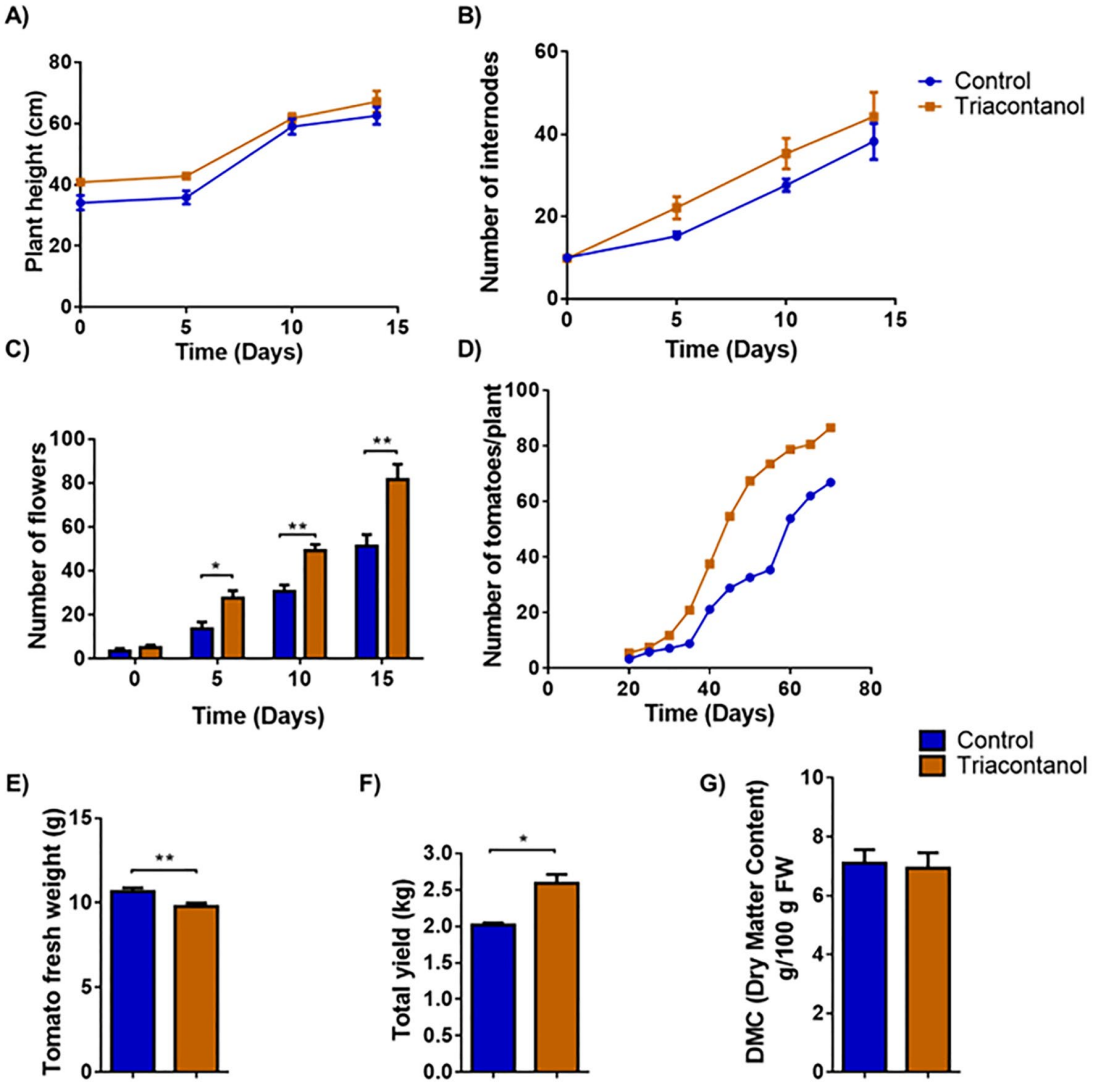
Phenotypical parameters and fruit yield of triacontanol-biostimulated and control plants. Four-week plants were biostimulated weekly with a foliar application of 70 µM triacontanol. (**A**) plant height, (**B**) number of internodes, (**C**) number of flowers of biostimulated plants, measured 15 days after the first biostimulation. (**D**) Number of fruits per plant, determined starting from day 20 after the first biostimulation. (**E**) mean fresh weight, (**F**) total yield, (**G**) dry matter content (g/100 fresh weight) of fruits at harvest. Error bars are s.e.m. of three independent experiments. For each experiment, eight plants/group were analyzed. * *p* < 0.05, ** *p* < 0.01, by Student’s t-test.

The experiments were conducted in a hydroponic drip system, where tomato plants were grown according to the planned timelines, as reported in the Methods section. This study used the Minibel variety, which has a relatively rapid life cycle. To test the effects of triacontanol on tomato growth and development, plants were grown for 4 weeks and then biostimulated with foliar spraying of 70 µM triacontanol once a week until day 75. Starting from the first application, different phenological traits, such as plant height, number of internodes and flowers, as well as number of fruits and time of their development, were evaluated every five days. Treatment with the biostimulant did not affect the height of tomato plants (Fig. 1A) or the number of corresponding internodes (Fig. 1B). On the other hand, as reported in Fig. 1C, triacontanol biostimulation determined a significant increase in the number of flowers from 2 weeks after treatment (50.5% after 5 days, 37.6% after 10 days, and 37.3 after 15 days). The fruit set was followed until day 75 after the first triacontanol biostimulation (Fig. 1D). Starting from day 35 after the first application, the treatment determined a significant increase in the number of fruits (+ 57.7%), to decline until day 75 (+ 22.8%), when red ripe fruits were harvested. Harvested fruits were weighed individually. The average weight of fruits from biostimulated plants was 16% lower than that from untreated ones (Fig. 1E); accordingly, the total yield of harvested tomatoes was 28% higher in biostimulated plants (Fig. 1F). Finally, the water/dry matter ratio did not differ significantly between tomatoes from biostimulated and untreated plants (Fig. 1G).

### Quality traits of tomato fruits from triacontanol-biostimulated plants

**Figure 2:**
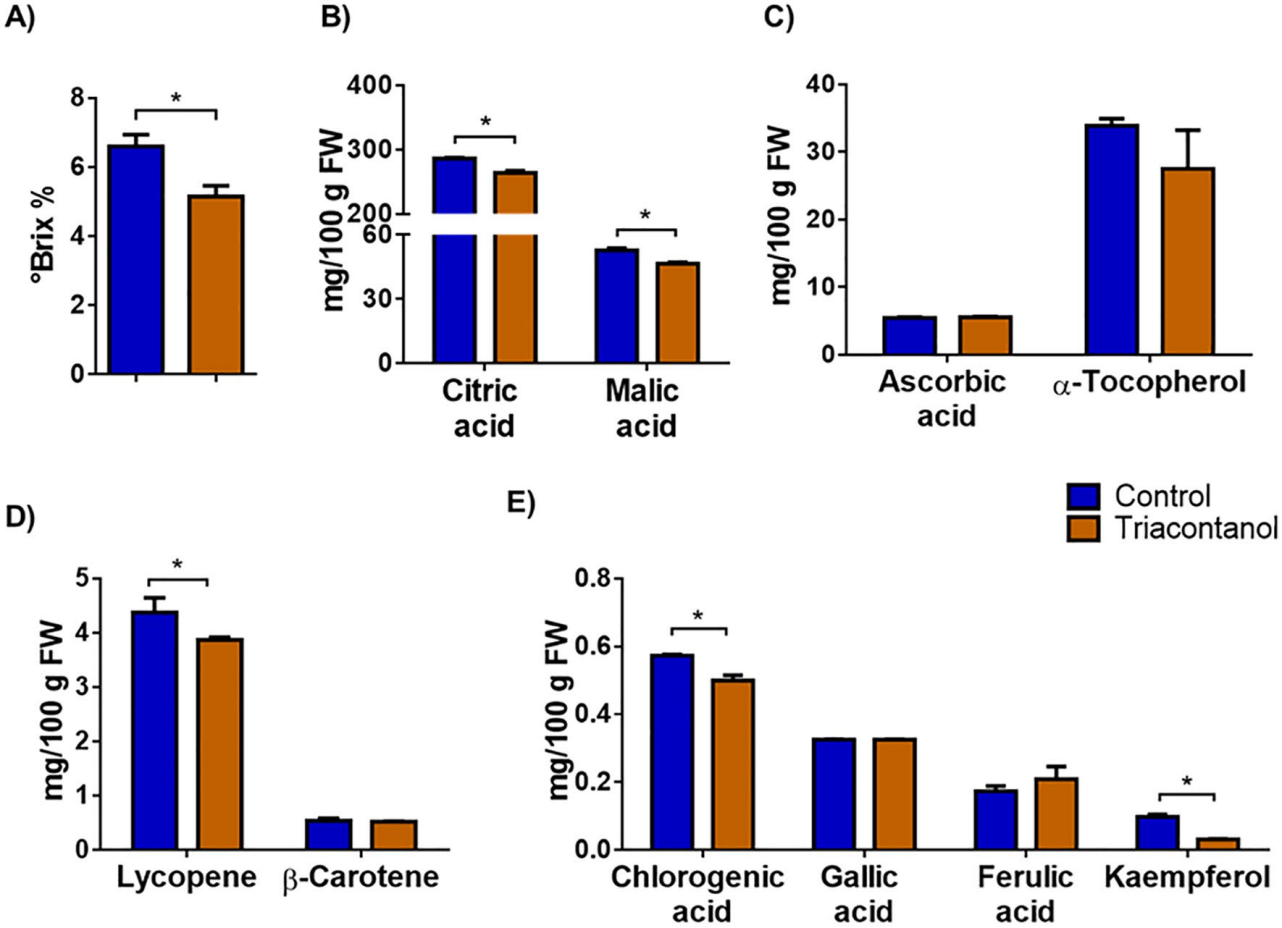
Biochemical characterization of fruits from triacontanol-biostimulated and control plants . (**A**) SSC, expressed in °Brix, of tomato juice extracted from fruits. Content of (**B**) organic acids, (**C**) vitamins, (**D**) carotenoids, (**E**) polyphenols, as estimated by HPLC analysis. Error bars are s.e.m. of three independent experiments. For each experiment, nine tomato/group were analyzed. * *p* < 0.05, by Student’s t-test.

Biochemical characterization of the fruits was carried out to test whether triacontanol biostimulation also affected the quality traits of tomatoes. We first measured the soluble solid content (SSC)^18^. SSC, expressed in °Brix, represents the content of sugars and organic acids, the main components contributing to fruit quality^19^. Upon triacontanol biostimulation, the °Brix value of fruits decreased from 6.6 to 5.2 (Fig. 2A). We also analyzed citric and malic acids, the main organic acids in tomato fruits. As shown in Fig. 2B, levels of both acids were slightly reduced by triacontanol treatment.

We also analyzed the content of vitamins, carotenoids, and polyphenols, which significantly contribute to the nutritional value of tomato. Levels of ascorbic acid (vitamin C) and α-tocopherol, the main form of vitamin E in tomatoes^20^, were not affected by triacontanol treatment (Fig. 2C). Lycopene and β-carotene account for about 90% and 10% of total carotenoids in tomato fruits, respectively, representing the main antioxidant compounds^21^. The lycopene content was slightly reduced (− 11%) in fruits from triacontanol-stimulated plants, whereas β-carotene levels were unaffected (Fig. 2D). Polyphenols also contribute to antioxidant and free radical-scavenging activities in fruits. Accordingly, we determined the concentration of gallic, chlorogenic, and ferulic acids, as well as that of kaempferol, as representatives of the main classes of phenolics. As shown in Fig. 2E, triacontanol biostimulation slightly reduced the levels of chlorogenic acid (− 13%). On the contrary, the concentration of gallic and ferulic acids was unaffected upon triacontanol treatment. Instead, a more significant reduction (− 68%) of kaempferol was observed.

These biochemical analyses revealed that biostimulation with triacontanol partially alters the nutritional properties of the tomatoes, slightly reducing the SSC content and the levels of some molecules having antioxidant activity. However, given the minimal changes observed, these data de facto do not underline substantial differences in the nutritional quality of the fruits from biostimulated plants.

### Analysis of germination and hormone levels of triacontanol-treated seeds

**Figure 3:**
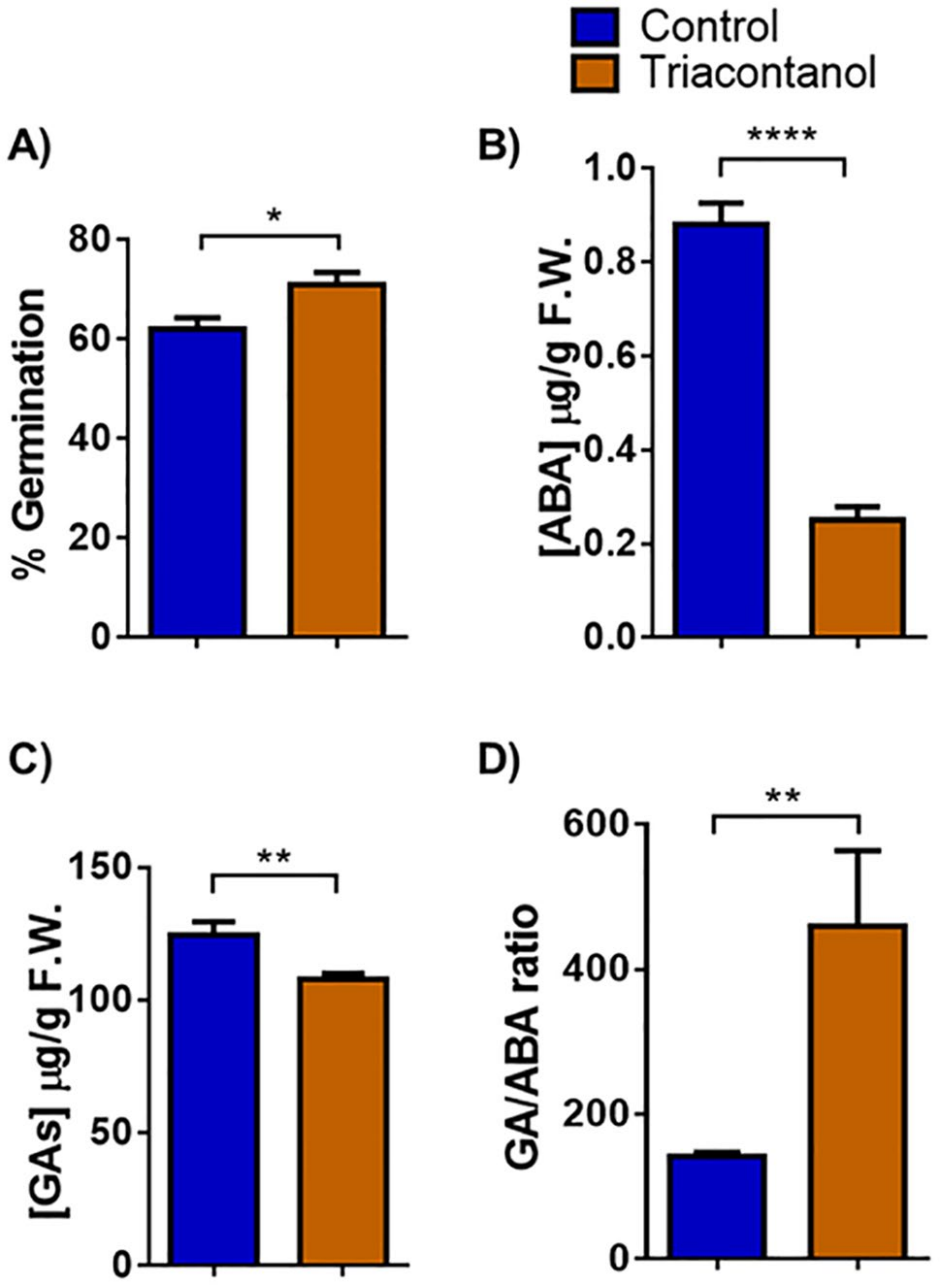
Effect of triacontanol priming on seed germination. **(A**) Germination rate was determined on 200 tomato seeds soaked in 70 µM triacontanol or water for 4 days and placed in a small-scale hydroponic system. The seeds’ germination rate was assessed after one week. ABA (**B**) and GAs (**C**) levels in triacontanol- and water-primed seeds, as estimated by HPLC analysis. (**D**) GAs/ABA ratio. Error bars are s.e.m. of three independent experiments. * *p* < 0.05, ** *p* < 0.01, **** *p* < 0.0001, by Student’s t-test.

Very recently, it has been reported that seed treatment with triacontanol increases the germination rate in *Phaseolus vulgaris*^22^. Accordingly, we tested the effect of triacontanol priming on tomato seed germination. For this purpose, seeds were treated with 70 µM triacontanol for 4 days before being sowed in a hydroponic system. As shown in Fig. 3A, seed priming slightly increased the germination rate. Since seed germination results from the fine-tuning regulation of a wide range of signals, including the ratio between abscisic acid (ABA) and gibberellins (GAs)^23^, an HPLC analysis of ABA and GAs was conducted to investigate whether triacontanol treatment alters hormone levels. Triacontanol application strongly decreased the amount of ABA in the seed (− 63%, Fig. 3B), whereas that of GAs was slightly reduced (− 16%, Fig. 3C). Consequently, the ABA/GAs ratio was significantly lowered (Fig. 3D), thus suggesting that an alteration of hormone homeostasis can account for the positive effect of triacontanol on seed germination.

### Biochemical response of triacontanol-treated plants to salt stress

**Figure 4:**
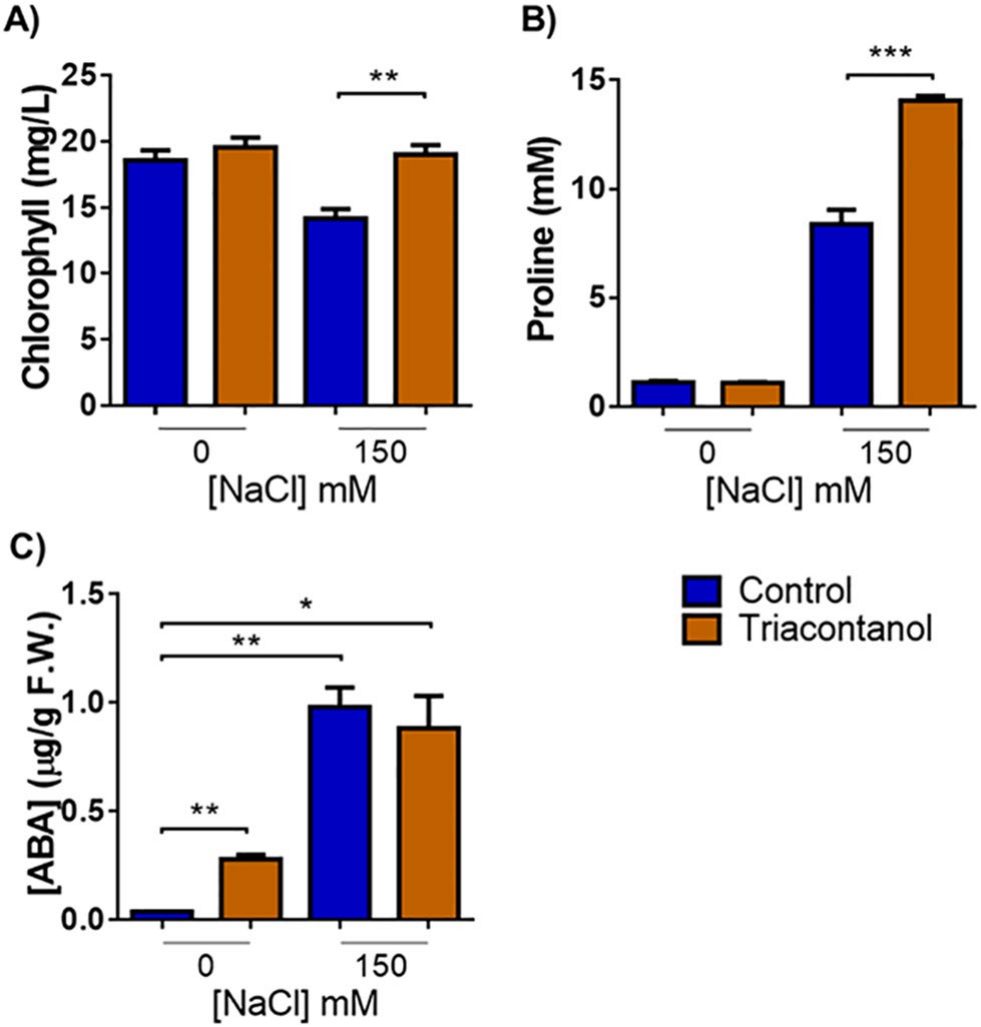
Triacontanol-biostimulation increases tolerance to salt stress. (**A**) Chlorophyll, (**B**) proline, and (C) ABA content determined on detached leaves of two-month-old tomato plants treated with a solution of 150 mM NaCl every other day for two weeks. Error bars are s.e.m. of three independent experiments. For each experiment, eight plants/group were analyzed. * *p* < 0.05, ** *p* < 0.01, *** *p* < 0.001, by Student’s t-test.

Recent information proposes a protective effect of triacontanol against abiotic stresses in different species. In order to ascertain whether triacontanol was able to improve abiotic stress resistance in tomato, biostimulated and control plants were subjected to saline stress and further analyzed for different biochemical parameters. For example, chlorophyll (Chl) depletion is a typical well-described process occurring in plants under salinity conditions, representing a good indicator of stress-induced damage^24^. Therefore, total Chl was determined spectrophotometrically in leaves of two-month-old tomato plants challenged with salt stress and biostimulated with triacontanol. As shown in Fig. 4A, salt stress significantly reduced (− 28%) the amount of total Chl in stressed control plants, whereas no significant Chl depletion was observed in the triacontanol-treated ones.

Osmotic imbalance and sodium toxicity are distinctive effects of salt stress^25^. Plants activate different mechanisms to limit the cellular damage induced by high salinity, including the synthesis of compatible osmolytes, mainly proline^26^. Since it is well-known that proline accumulation under salt stress is correlated with stress tolerance, an increased amount of this osmolyte upon biostimulation indicates the activation of mechanisms leading to salt stress resistance^26^. As shown in Fig. 4B, triacontanol treatment did not alter the proline content in biostimulated plants grown without stress. Conversely and as expected, saline stress triggered a substantial accumulation of proline in plants; the increase of this amino acid was higher in triacontanol-treated plants than in control ones.

Abscisic acid (ABA) is the primary hormone in abiotic stress responses^27^. The increase in ABA levels, induced following exposition to high salinity conditions, is essential for plant tolerance acquisition^28^. Figure 4C shows that salt stress induced a similar increase of ABA both in control and triacontanol-treated plants. However, it is noteworthy that triacontanol caused a significant rise in ABA without stress, indicating that biostimulation per se could represent a mild stress signal that might confer a basal protection toward external challenges by activating ABA-dependent tolerance mechanisms.

Overall, these results demonstrate the ability of triacontanol to mitigate salt-induced damage by activating typical plant response mechanisms toward excess salinity.

### Proteomic analysis of leaves and fruits from triacontanol-biostimulated plants

The effects on phenotype, fruit yield, and metabolite levels in response to triacontanol treatment prompted us to investigate the molecular mechanisms underlying biostimulation. To this end, a TMT-based proteomic investigation was conducted on both leaves and fruits from triacontanol-treated and control plants. Fully developed leaves or fully mature fruits from the last two clusters were harvested at day 75 after the first biostimulation (103-day-old plants) and were homogenized in liquid N_2_. TCA-acetone precipitated proteins were then subjected to proteomic analysis, as described in the experimental section. All peptides and proteins identified in tomato leaves and fruits are reported in Supplementary Tables S1 and S2, respectively. In leaves, 5025 proteins were identified and quantified in triacontanol-treated and control plants. Considering a fold change value ≥ 1.3, 43 differentially represented proteins (DRPs) were ascertained, of which 22 were over-represented and 21 were down-represented (Table 1).

**Table 1:** Differentially represented proteins identified in leaves of tomato plants treated with triacontanol.

Accession	Gene name after BlastP search	Description	Sum PEP score	Peptides (number)	PSMs	Mascot score	Abundance ratio: (treated)/(control)
Down-represented proteins							
A0A3Q7IJL2	LOC101266879	Uncharacterized protein—Bifunctional inhibitor/plant lipid transfer protein/seed storage helical domain-containing protein	26.596	3	37	1432	0.516
Q05538	CHI9	Basic 30 kDa endochitinase	68.365	6	76	2445	0.533
A0A3Q7E938	543986	Uncharacterized protein—Glucan endo-1,3-beta-D-glucosidase	89.721	10	57	1442	0.561
A0A3Q7G3L7	LOC101254763	60S ribosomal protein L36	7.706	3	7	81	0.591
A0A3Q7GUG2	LOC101264431	60S ribosomal protein L36	10.501	3	8	199	0.620
A0A3Q7I5D6	101258992	40S ribosomal protein S24	28.692	5	18	490	0.643
Q05539	CHI3	Acidic 26 kDa endochitinase	91.702	8	67	1907	0.648
R9R6F4	PHS1	Terpene synthase 20	26.575	9	18	315	0.673
A0A3Q7H8G2	LOC101252200	Uncharacterized protein—Cysteine proteinase 3	137.002	11	172	4601	0.704
A0A3Q7JCE2	LOC107002472	Uncharacterized protein—CCHC-type domain-containing protein	16.112	3	12	346	0.705
P32045	PR-P2	Pathogenesis-related protein P2	39.617	6	45	1162	0.714
A0A3Q7GZ83	101251893	60S ribosomal protein L27	18.618	4	18	347	0.728
A0A3Q7IDU2	101254713	AAI domain-containing protein—bifunctional inhibitor/plant lipid transfer protein/seed storage helical domain-containing protein	16.596	3	34	543	0.731
O65818	H2B-2	Histone H2B.2	58.14	8	105	2059	0.734
A0A3Q7HYX4	101249118	TRASH domain-containing protein	18.29	3	13	243	0.738
P28032	ADH2	Alcohol dehydrogenase 2	6.546	2	3	53	0.746
A0A3Q7ILR1	LOC101266177	TRASH domain-containing protein	24.027	4	17	331	0.746
A0A3Q7HN20	VPE3	Uncharacterized protein	83.068	12	62	1290	0.748
A0A3Q7ET47	101265851	LRRNT_2 domain-containing protein	15.118	2	11	257	0.762
A0A3Q7J606	LOC101265775	Phytocyanin domain-containing protein	27.336	5	22	565	0.763
K4CWS6	UGT75C1	UDP-glycosyltransferase 75C1	49.617	9	36	954	0.765
Up-represented proteins							
A0A3Q7GNX4	101256678	Non-specific lipid-transfer protein	9.962	3	12	312	2.331
A0A3Q7J9T5	101247110	Uncharacterized protein—methyltransferase type 11 domain-containing protein	7.508	3	6	88	2.069
I3QHF0	LOC543955	Proteinase inhibitor II	39.968	7	38	958	1.759
A0A6F8PJG0	23DOX	Alpha-tomatine 23-hydroxylase	7.706	2	4	157	1.690
Q9LEG1	cathDInh	Cathepsin D inhibitor	12.005	3	8	199	1.536
A0A3Q7FP33	cathDInh (precursor)	Uncharacterized protein—Cathepsin D inhibitor	15.364	4	9	151	1.533
A0A3Q7IPA2	101243656	Phenylalanine ammonia-lyase	45.495	9	28	559	1.501
A0A3Q7GLC6	LOC101261413	Methylenetetrahydrofolate reductase	27.013	7	21	374	1.475
A0A3Q7HNV7	LOC101258288	Mannan endo-1,4-beta-mannosidase	11.009	4	8	123	1.453
P93220	ER5	Ethylene-responsive late embryogenesis-like protein	7.634	2	4	69	1.445
A0A3Q7HHV8	LOC101268414	Uncharacterized protein—Exoribonuclease phosphorolytic domain-containing protein	5.392	2	3	56	1.376
A0A3Q7HD77	LOC101264947	Uncharacterized protein—Cytochrome P450	8.658	3	9	128	1.360
A0A3Q7H1D7	101248584	Eukaryotic translation initiation factor 3 subunit C	23.202	6	15	282	1.351
A0A3Q7HCE6	LOC101255510	Uncharacterized protein—glycosyltransferase 2 family protein	66.756	16	71	1693	1.347
Q9M4X2	LOC100736434	Putative cytochrome P450	70.937	13	46	1177	1.342
A0A0C6G3Q8	SSR2	Sterol side chain reductase	102.479	20	86	1570	1.334
A0A3Q7IIH1	101261972	Mannan endo-1,4-beta-mannosidase	16.752	4	13	293	1.332
Q40144	XTH1	Probable xyloglucan endotransglucosylase/hydrolase 1	33.349	7	25	553	1.322
A0A3Q7JR76	101245329	Uncharacterized protein—Gnk2-homologous domain-containing protein	91.96	14	66	1813	1.319
A0A3Q7J4D7	101266797	SRP54 domain-containing protein	5.081	3	4	64	1.314
A0A3Q7I868	101247557	Uncharacterized protein—Wound-induced proteinase inhibitor 1	34.852	3	26	1094	1.308
A0A3Q7G4N5	LOC101251468	Uncharacterized protein—DUF4057 domain-containing protein	28.371	5	14	444	1.304

Accession code, gene name following BlastP searching, protein description, sum PEP score, number of identified peptides, peptide spectrum matches, Mascot score value, and abundance ratio value (triacontanol vs. control) are reported. Identification and quantification details are described in Supporting Information Table S1.

As far as fruits, 3336 proteins were identified and quantified in triacontanol-treated and control plants. Considering the same fold change value reported above, 25 DRPs were assigned, of which 16 were over-represented and 9 were down-represented (Table 2). Pathogenesis-related protein P2, 60S ribosomal protein L27, and some glycosyl transferase isoforms were commonly differentially represented in tomato leaves and fruits after triacontanol treatment.

**Table 2:** Differentially represented proteins identified in berries of tomato plants treated with triacontanol.

Accession	Gene name after BlastP search	Description	Sum PEP Score	Peptides (number)	PSMs	Score Mascot	Abundance ratio: (treated)/(control)
Down-represented proteins							
P32045	PR-P2	Pathogenesis-related protein P2	83.736	8	74	1866	0.601
A0A3Q7FQ22	LOC101268268	Uncharacterized protein—ZZ-type domain-containing protein	68.917	13	39	872	0.638
A0A3Q7F9I5	101252242	Uncharacterized protein	12.451	5	11	186	0.697
A0A3Q7H1A3	LOC101248885	PHB domain-containing protein	48.562	11	33	586	0.708
A0A3Q7IGG9	LOC101245896	Uncharacterized protein—DUF547 domain-containing protein—putative cytochrome P450	6.782	2	2	43	0.721
O24032	GPXle-2	Glutathione peroxidase	15.917	3	9	144	0.739
A0A3Q7G391	101257258	Uncharacterized protein	7.269	2	2	34	0.751
A0A3Q7EYY4	LOC101254660	FYVE-type domain-containing protein	16.861	3	6	167	0.755
A0A3Q7GGC9	LOC101055568	CG-1 domain-containing protein	6.637	2	3	50	0.762
Up-represented proteins							
A0A3Q7JGP1	LOC101253952	Tubulin beta chain	73.786	14	61	1150	1.563
A0A3Q7GNF0	101E + 08	Usp domain-containing protein	38.127	3	41	1218	1.472
A0A3Q7F7I3	ACO4	Fe2OG dioxygenase domain-containing protein	23.62	6	27	423	1.456
A0A3Q7JHJ2	101E + 08	Uncharacterized protein—40S ribosomal protein S18	34.109	5	27	598	1.445
A0A3Q7IJX7	101E + 08	Uncharacterized protein—Histidine decarboxylase	218.428	18	178	4596	1.417
A0A3Q7HH84	gtsatom	Uncharacterized protein—UDP-glycosyltransferases domain-containing protein	29.178	4	14	290	1.409
A0A3Q7HU74	LOC101266248	Tr-type G domain-containing protein	40.494	9	51	730	1.379
A0A3Q7HHL0	101E + 08	Tubulin alpha chain	155.333	17	86	2522	1.378
A0A3Q7H2Z1	101E + 08	Uncharacterized protein—Agmatine coumaroyltransferase-2-like	10.17	2	6	99	1.378
A0A3Q7GZ83	101E + 08	60S ribosomal protein L27	19.902	5	12	207	1.374
A0A3Q7HCR0	101E + 08	60S ribosomal protein L27	26.963	5	17	270	1.356
A0A3Q7FZ65	101E + 08	Bet_v_1 domain-containing protein	101.82	8	142	3066	1.345
A0A3Q7I9U4	SAHH	Adenosylhomocysteinase	420.378	33	554	12939	1.342
A0A3Q7JFH7	101E + 08	Uncharacterized protein—60S ribosomal protein L21	18.108	5	21	253	1.341
A0A3Q7JAB8	101E + 08	40S ribosomal protein S12 1	67.59	8	46	987	1.313
A0A3Q7F289	LOC101259704	Glycosyltransferase	18.974	5	12	308	1.311

Accession code, gene name following BlastP searching, protein description, sum PEP score, number of identified peptides, peptide spectrum matches, Mascot score value, and abundance ratio value (triacontanol vs. control) are reported. Identification and quantification details are described in Supporting Information Table S2.

**Figure 5:**
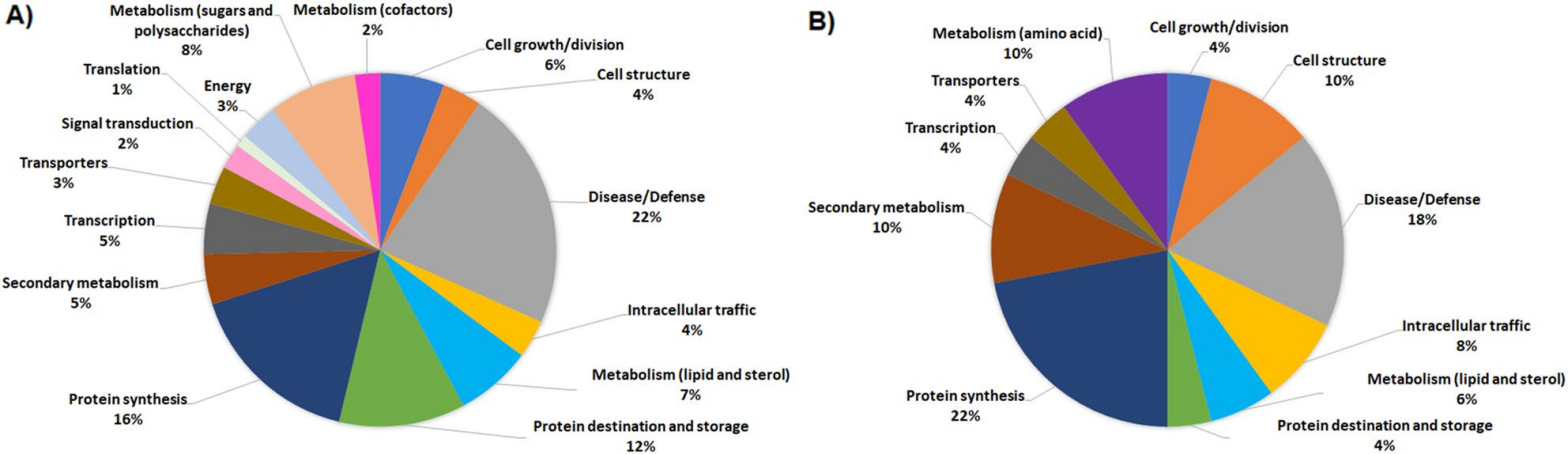
Functional distribution of differentially represented proteins in leaves (**A**) and fruits (**B**) of tomato following treatment with triacontanol. Identified protein species were initially assigned with Mercator software, followed by a functional group cataloguing also including information from literature data. When a protein showed multiple functions, each functional contribution was calculated to have a final cumulative value equal to 1.

DRPs were at first indexed through an initial functional assignment obtained from analysis with both Mercator software^29^ and information from recent scientific literature, which were integrated and used to finally classify proteins according to Bevan functional cataloguing^30^ (Supplementary Tables S3 and S4). This approach attributed a function to all leaf and fruit DRPs. According to their identity (and incidence > 5%), leaf DRPs were related to the functional categories of (i) disease/defense (22.2%); (ii) protein synthesis (16.3%); (iii) protein destination and storage (11.6%); (iv) metabolism (sugars and polysaccharides) (8.1%); (v) metabolism (lipids and sterols) (7.0%); (vi) cell growth/division (5.9%), underlining the prominent molecular mechanisms modified in tomato leaves after triacontanol treatment (Fig. 5A). Similarly, fruit DRPs were related to the functional groups of (i) protein synthesis (22.0%); (ii) disease/defense (18.0%); (iii) cell structure (10.0%); (iv) metabolism (amino acids) (10.0%); (v) secondary metabolism (10.0%); (vi) intracellular traffic (8.0%); (vii) metabolism (lipids and sterols) (6.0%), highlighting the prominent molecular pathways affected in tomato fruits as a result of triacontanol action on plants (Fig. 5B).

**Figure 6:**
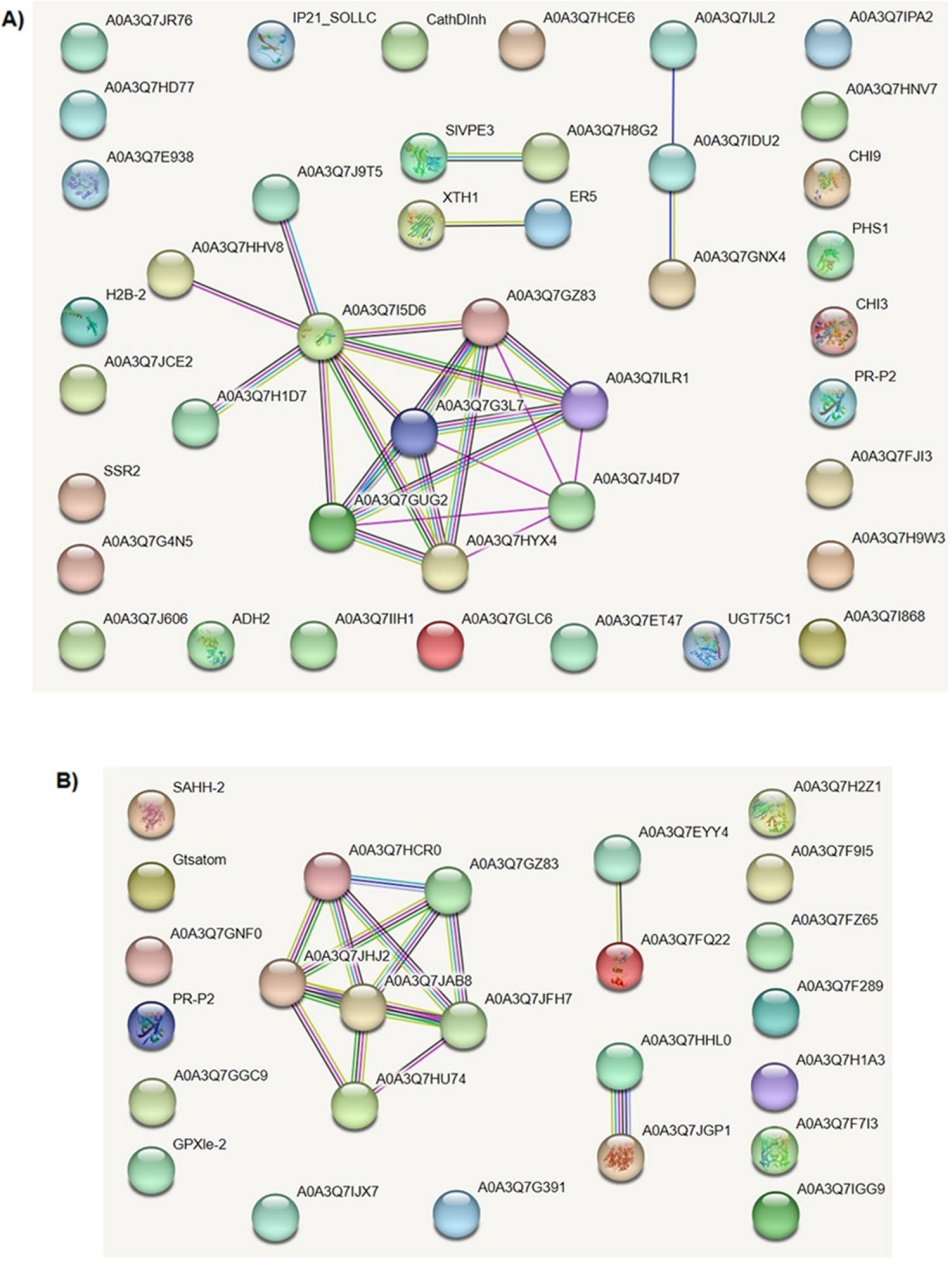
STRING analysis of DRPs identified in tomato leaves (**A**) and fruits (**B**) after triacontanol treatment. The protein interaction network of the above-mentioned DRPs (43 and 25, respectively) was assigned according to medium-confidence interactions (0.4). Functional protein associations were based on the corresponding data recorded in the STRING database. Panel A, A0A3Q7JR76, uncharacterized protein-Gnk2-homologous domain-containing protein; A0A3Q7HD77, uncharacterized protein-cytochrome P450; A0A3Q7E938, uncharacterized-protein-glucan endo-1,3-beta-D-glucosidase; H2B-2, histone H2B.2; A0A3Q7JCE2, uncharacterized protein-CCHC-type domain-containing protein; SSR2, sterol side chain reductase; A0A3Q7G4N5, uncharacterized protein-DUF4057 domain-containing protein; A0A3Q7J606, phytocyanin domain-containing protein; ADH2, alcohol dehydrogenase 2; A0A3Q7IIH1, mannan endo-1,4-beta-mannosidase; A0A3Q7GLC6, methylenetetrahydrofolate reductase; A0A3Q7ET47, LRRNT-2 domain-containing protein; UGT75C1, UDP-glycosyltransferase 75C1; A0A3Q7I868, uncharacterized protein-wound-induced proteinase inhibitor 1; A0A3Q7H9W3, Putative cytochrome P450; A0A3Q7FJI3, alpha-tomatine 23-hydroxylase; PR-P2, pathogenesis-related protein P2; CHI3, acidic 26 kDa endochitinase; PHS1, terpene synthase 20; CHI9, basic 30 kDa endochitinase; A0A3Q7HNV7, mannan endo-1,4-beta-mannosidase; A0A3Q7IPA2, phenylalanine ammonia-lyase; A0A3Q7IJL2, uncharacterized protein-bifunctional inhibitor/plant lipid; A0A3Q7IDU2, AAI domain-containing protein; A0A3Q7GNX4, non-specific lipid-transfer protein; XTH1, probable xyloglucan endotransglucosylase/hydrolase 1; ER5, ethylene-responsive late embryogenesis-like protein; A0A3Q7H8G2, uncharacterized protein-cysteine proteinase 3; SIVPE3, transfer protein/seed storage helical domain-containing protein; A0A3Q7J9T5, uncharacterized protein-methyltransferase type 11 domain-containing protein; A0A3Q7HHV8, uncharacterized protein-exoribonuclease phosphorolytic domain-containing protein; A0A3Q7I5D6, 40S ribosomal protein S24; A0A3Q7H1D7, eukaryotic translation initiation factor 3 subunit C; A0A3Q7GZ83, 60S ribosomal protein L27; A0A3Q7G3L7, 60S ribosomal protein L36; A0A3Q7ILR1, TRASH domain-containing protein; A0A3Q7GUG2, 60S ribosomal protein L36; A0A3Q7HYX4, TRASH domain-containing protein; A0A3Q7J4D7, SRP54 domain-containing protein; A0A3Q7HCE6, uncharacterized protein-glycosyltransferase 2 family protein; CathDInh, uncharacterized protein-cathepsin D inhibitor; IP21_SOLLC, wound-induced proteinase inhibitor 2. Panel B. SAHH-2, adenosylhomocysteinase; Gtsatom, uncharacterized protein-UDP-glycosyltransferases domain-containing protein; A0A3Q7GNF0, Usp domain-containing protein; PR-P2, pathogenesis-related protein P2; A0A3Q7GGC9, CG-1 domain-containing protein; GPXle-2, glutathione peroxidase; A0A3Q7IJX7, uncharacterized protein-histidine decarboxylase; A0A3Q7JHJ2, uncharacterized protein-40S ribosomal protein S18; A0A3Q7GZ83, 60S ribosomal protein L27; A0A3Q7HCR0, 60S ribosomal protein L27; A0A3Q7JFH7, uncharacterized protein-60S ribosomal protein L21; A0A3Q7JAB8, 40S ribosomal protein S12 1; A0A3Q7HU74, Tr-type G domain-containing protein; A0A3Q7EYY4, FYVE-type domain-containing protein; A0A3Q7FQ22, uncharacterized protein-ZZ-type domain-containing protein; A0A3Q7HHL0, tubulin alpha chain; A0A3Q7JGP1, tubulin beta chain; A0A3Q7H2Z1, uncharacterized protein-agmatine coumaroyltransferase-2-like; A0A3Q7F9I5, uncharacterized protein; A0A3Q7FZ65, Bet_v_1 domain-containing protein; A0A3Q7IGG9, uncharacterized protein-DUF547 domain-containing protein; A0A3Q7H1A3, PHB domain-containing protein; A0A3Q7F7I3, Fe2OG dioxygenase domain-containing protein; A0A3Q7F289, glycosyltransferase; A0A3Q7G391, uncharacterized protein.

STRING protein interaction analysis^31^ of triacontanol-associated leaf DRPs showed that 39.5% of them are functionally linked; four networks were identified as not linked to each other, among which the biggest one included 10 proteins (23.3% of the total) (Fig. 6A). This finding suggested the absence of a unique functional assembly bridging different components from various deregulated metabolic pathways related to triacontanol treatment; conversely, it emphasized that triacontanol exerts its action on distinct molecular processes. This condition substantially did not change whether the more investigated *Arabidopsis thaliana* STRING interaction database was searched instead of the tomato counterpart (data not shown). Similarly, STRING analysis of fruit DRPs showed that 40.0% of them are functionally linked; three networks were identified as not linked to each other, among which the biggest one included 6 proteins (24.0% of the total) (Fig. 6B). This finding again suggested the absence of a unique functional assembly linking together different components from various deregulated molecular processes associated with triacontanol treatment; conversely, it emphasized that the biostimulant elicits its action on independent pathways.

## Discussion

Although different studies proved that triacontanol exogenous administration promotes the growth and yield of many plants, its effects and mechanism of action still need a detailed characterization. A better understanding of the action of triacontanol is highly desirable in terms of deepening scientific knowledge of plant growth regulation and improving crop yield and quality. Therefore, this work aimed to characterize the effect of triacontanol on tomato plant growth and productivity. The choice of tomato is because it is a model species for plant biochemistry and genetic studies and it is one of the most relevant crops for human nutrition. Although the growth-stimulating effect of triacontanol has been demonstrated in different species, detailed phenotypical data are very scarce, particularly in tomato, where only an increase in plant dry weight and leaf area have been reported^32^. The analysis of phenological traits reported in the present study has demonstrated that foliar applications of 70 µM triacontanol to Minibel tomato resulted in advanced blooming and an increased number of flowers and fruits. In contrast, neither plant height nor internode number was affected. Analysis of fruits’ phenological and biochemical quality traits showed that fruits of biostimulated plants were smaller and contained slightly lower amounts of soluble solids, citric and malic acids, lycopene, and chlorogenic acid. The effects of triacontanol on seed germination were also studied. Triacontanol priming increased the germination rate by altering hormone homeostasis. Interestingly, a reduction in ABA levels and, consequently, in the ABA/GA ratio was observed. Triacontanol treatment also reduced salt stress-induced damage in mature plants, as revealed by the analysis of various stress-related parameters, such as proline, chlorophyll, and ABA, confirming the protective role of triacontanol in abiotic stress responses^9^.

The pleiotropic action of triacontanol made it challenging to address the question of its mechanism(s) of action. A TMT-based proteomic investigation was carried out on leaves and fruits to elucidate the biological processes targeted by triacontanol in the plant cell. In leaves, the analysis revealed forty-three DRPs, which were classified into different functional categories according to GO (Gene Ontology) annotation and literature analysis. Twenty-one of the forty-three DRPs were down-represented following triacontanol treatment. They could be grouped into two main functional classes. The largest group included proteins involved in resistance to biotic stress. Basic 30 kDa endochitinase (Q05538), glucan endo-1,3-β-D-glucosidase (A0A3Q7E938), acidic 26 kDa endochitinase (Q05539), pathogenesis-related protein PR2 (P332045) and putative cysteine proteinase 3 (A0A3Q7H8G2) are extracellular proteins with polysaccharide- or protein-degrading activities involved in the immune response of plants^33–35^. LRRNT_2 domain-containing protein (A0A3Q7HN20) is a membrane receptor involved in the defense against fungi^36^. Terpene synthase 20 (R9R6F4) is an enzyme involved in the biosynthesis of volatile signaling defense monoterpenes, whose gene is up-regulated in tomato upon pest infection^37^. UDP-glycosyltransferase 75C1 (K4CWS6) is a member of the wide class of UDP-glycosyltransferases (UGTs) involved in the detoxification of xenobiotics and defense against pathogens^38^ and abiotic stresses^39^. The second group included two 60S, one 40S, and one 27S ribosomal proteins, which regulate protein synthesis by participating in the formation of ribosomal initiation complexes^40^, and two TRASH domain-containing proteins, which are requested for the functional integrity of ribosomes^41^. Other down-regulated proteins were miscellaneous or uncharacterized. From the above-reported data, it can be inferred that the triacontanol down-regulatory effect impacted mainly proteins involved in the defense from pathogens and protein synthesis.

Out of the twenty-two proteins that were over-represented in leaves after triacontanol treatment, eight were uncharacterized (A0A3Q7G4N5, A0A3Q7I868, A0A3Q7JR76, A0A3Q7HCE6, A0A3Q7HD77, A0A3Q7HHV8, A0A3Q7FP33 and A0A3Q7J9T5). Their physiological function is far from being fully understood, and accordingly, further dedicated studies are needed to clarify their role in the triacontanol-plant interaction. The remaining proteins displayed a more dispersed distribution amongst functional classes with respect to the down-represented ones. This represented a further challenge in identifying the biological processes affected by triacontanol treatment in tomato. Nevertheless, four proteins were involved in the biosynthesis and metabolism of sterols, which influence different aspects of plant physiology, such as membrane biogenesis (sterols), growth and development, including flowering (brassinosteroids, BRs), and adaptation to abiotic stresses (BRs, steroidal glycoalkaloids)^42,43^. These proteins are sterol side chain reductase 2 (SSR2, A0A0C6G3Q8), α-tomatine 23-hydroxylase (A0A3Q7FJI3), and two putative cytochromes P450 (Q9M4X2 and A0A3Q7HD77). In tomato, two SSR1 and SSR2 homologs of the sterol side chain reductase occur. SSR1, corresponding to the DWF1 mutation that produces dwarf plants, reduces 24-methylenecholesterol to campesterol; SSR2 reduces the C24 double bond of Δ^24(25)^–sterols, converting cycloartenol to 24-methylenecycloartanol in the biosynthetic pathway of BRs^44^ and steroidal glycoalkaloids^45^. The most abundant glycoalkaloid in tomato is α-tomatine, which is accumulated in leaves and immature fruits as an antibiotic compound^46^. α-Tomatine, which has a bitter taste, is converted during ripening in the non-toxic compound esculeoside A by different enzymatic steps, including that catalyzed by α-tomatine 23-hydroxylase^46^. From the above-reported results, it is tempting to speculate that an over-representation of enzymes involved in the steroid pathway occurs in the leaves of triacontanol-stimulated plants, which might be related to the phenological data showing increased flowering and fruit yield upon triacontanol administration. Further dedicated studies are needed to validate this hypothesis.

In parallel, three additional cell wall polysaccharides-modifying enzymes were also over-represented proteins in the leaves of triacontanol-treated plants, namely two mannosidases (A0A3Q7IIH1 and A0A3Q7HNV7) and a xyloglucan endotransglucosylase (Q40144). Their increased levels were associated with the physiological need of the plant to remodel the cell wall during organism growth. A similar augmented quantitative trend was also observed for proteins belonging to the group of components involved in stress response, namely phenylalanine ammonia-lyase (PAL, A0A3Q7IPA2) and ethylene-responsive late embryogenesis-like protein (ER5, P93220). PAL is the key regulatory enzyme of phenylpropanoid metabolism, playing a pivotal role in stress resistance^47^. ER5 belongs to the large group of LEA proteins involved in response to abiotic stress, particularly salt and drought stress^48^. Therefore, the over-representation of the latter two proteins well correlates with the observed increased resistance of plants treated with triacontanol towards salt stress.

In fruits, proteomic analysis revealed twenty-five DRPs, among which nine were down-regulated and sixteen up-regulated. Four of the down-represented proteins were uncharacterized, while three had uncertain functions. The remaining two were stress-related proteins, namely pathogenesis-related protein P2 (PR-P2, P32045), and glutathione peroxidase (GPxle-2, O24032).

Sixteen DRPs were over-represented in tomato fruits. The largest group included five members, corresponding to 60S (A0A3Q7HCR0, A0A3Q7GZ83, and A0A3Q77JFH7) and 40S (A0A3Q7JAB8 and A0A3Q7JHJ2) ribosomal proteins, which regulate protein synthesis by participating in the formation of ribosomal initiation complexes^40^. Additional augmented components were two proteins belonging to the family of UDP-glycosyltransferases, which function primarily in detoxifying xenobiotics but are also involved in the defense against pathogens^38^ and toward abiotic stresses^39^. Tubulin α-(A0A3Q7HHL0) and β-chains (A0A3Q7JGP1) were also identified as over-represented DRPs. In the latter context, it has already been shown that the synthesis of structural proteins like tubulins increases during maximal fruit growth in correlation to the morphological alterations underlying fruit growth and ripening. Adenosylhomocysteinase (A0A3Q719U4), involved in the regeneration of S-adenosyl-L-methionine^49^, three polypeptides containing specific domains but of uncertain functions, and two uncharacterized proteins completed the list of over-represented proteins in the fruits from triacontanol-treated plants.

Overall, it can be concluded that the proteomic analysis of fruits was less informative than that of leaves. Most of the proteins were uncharacterized; thus, it was only possible to assign them to functional classes according to Bevan functional cataloguing, without being able to infer a specific function. Since their physiological role is far from being established, future dedicated investigations are needed to clarify their specific function in the triacontanol-plant interaction. On the other hand, proteomic results, showing that triacontanol did not significantly affect fruits’ protein repertoire, corroborate the substantial equivalence regarding nutritional properties between fruits from control and biostimulated plants.

In conclusion, data reported in this study reveal that triacontanol biostimulation, although not affecting tomato plant growth, promoted blooming and increased fruit yield, minimally altering the metabolic profile of fruits. Moreover, triacontanol biostimulation prevented the negative effects caused by salt stress. These effects were substantiated by the proteomic analysis, demonstrating that, in leaves, triacontanol increased the levels of proteins linked to development and abiotic stress response, simultaneously decreasing those implicated in biotic stress resistance. On the other hand, the proteome of fruits was only slightly altered by triacontanol treatment, explaining the equivalence of the nutritional properties of the fruits. Overall, our research provides evidence of how triacontanol affects growth, development, and stress resistance, contributing to shed some light on its mode of action, and paving the way for future studies for widespread exploitation of triacontanol in agricultural practices.

## Methods

### Plant material and stress conditions

Tomato plants (*S. lycopersicum* L., var. Minibel) seeds were purchased from JohnsonsTM (Kentford, United Kingdom). The Minibel tomato is a determinate-growing variety producing small-sized fruits and well suited for laboratory-scale cultivation. Seeds were grown in 11 l-pots in a hydroponic drip system on CocoPerlite 70/30 hydroponic substrate (Gold Label, Krommenie, NL). Pots were located on 80 l ponds filled with the Masterblend Kit solution (Masterblend International, Morris, IL, USA), according to the manufacturer’s instructions. Plants were cultivated in a growth chamber at 25 °C, under a 16/8 light/dark cycle, with a 200 µmol m^−1^ s^−1^ irradiation, as already described^50^. After four weeks, 70 µM triacontanol (Xi’an Neo Biotech Co., Xi’an, CHN) or water (control) was applied using bottles with a spray nozzle on tomato leaves (20 ml/plant) weekly until the complete fruit development.

For salt stress treatments, tomato plants were grown in universal soil for one month under the abovementioned conditions. Plants were then sprayed with 70 µM triacontanol^51^ or water (control) twice a week for one additional month. After the first four biostimulant applications, triacontanol-treated and control plants were treated with 150 mM NaCl on alternate days until the end of the experiment.

Experimental research, including the collection of plant material, complies with relevant institutional, national, and international guidelines and legislation.

### Analysis of plant phenotypes

Phenotypical analyses were performed on ten plants for each group. Selected plants were randomly distributed and considered for biological replicates. Plant height, internode number, and flower transition parameters were recorded on the first day of biostimulation and then every five days. The number of developing tomatoes per plant was assessed 20 days post-biostimulation and then collected every 5 days; the number, total yield, and fresh weight of red ripe fruits (stage VI)^52^ were assessed at harvest time. The tomato dry matter content was determined by dehydrating tomatoes in an oven at 70 °C until they reached a constant weight.

### Analysis of total soluble solids

The total soluble solids expressed as °Brix were determined at 20 °C on the supernatant generated by centrifuging the raw homogenate using a HI96800 Hanna Instruments (Woonsocket, RI, USA) digital refractometer.

### Extraction and analysis of organic acids and ascorbic acid

Extraction and analysis of organic acids from tomato fruits were performed according to Nour and colleagues^53^. Tomato fruits were freeze-dried and powdered using a blender. Briefly, 0.2 g of tomato powder was added to 6 ml of 25 mM potassium phosphate buffer, pH 2.5, and the suspension was vortexed for 10 min, at 25 °C. After centrifuging the mixture for 10 min at 3000 g, at 4 °C, the supernatant was cleared through a 0.2 µm filter prior to HPLC analysis.

The supernatant was analyzed with a Dionex Ultimate 3000 UHPLC system (Thermo Fisher Scientific, Waltham, MA, USA) with a Prevail C18 column (250 × 4.6 mm, 5 µm particle size) (Phenomenex, Torrance, CA, USA). The system comprises a quaternary LPG-3400RS pump, an autosampler WPS-3000, a column compartment TCC-3000, and a four-channel UV–Vis diode array detector 3000RS. The injection volume was set at 10 µl. Molecules were eluted isocratically with 25 mM potassium phosphate buffer, pH 2.5. The flow rate was 0.7 ml min^−1^, and the total run time was 15 min. The organic acids of interest were identified based on their retention time and UV spectra and by comparison with the standards of citric acid, malic acid, and L-ascorbic acid (Merck, Burlington, MA, USA). The purified standards were also used to establish calibration curves in the 1–10 mg/ml range for citric acid and 30–800 µg/ml range for malic acid at 215 nm wavelength, as well as in the 1.75–224 µg/ml range for L-ascorbic acid at 245 nm wavelength.

### Extraction and analysis of carotenoids and α-tocopherol

Extraction and analysis of carotenoids, and extraction of α-tocopherol from tomato powders were performed according to Fish and coworkers^54^ with some modifications. Briefly, 0.2 g tomato powder was dissolved by vortexing in deionized water (4 ml). Twenty-five ml of extraction mixture[hexane:acetone:ethanol, 2:1:1, 0.01% v/v butylated hydroxytoluene (BHT)] were added to the sample and, after 15 min of shaking, 4 ml of deionized water were added; then, the samples were stirred for 5 min. After centrifugation for 10 min at 2000 g, the clear upper orange-colored hexane phase was collected. The residue was extracted again, and the upper hexane layer was merged with the previous one. The extract was then evaporated using a rotary vacuum evaporator, and the concentrate was dissolved in 25 ml of sample buffer consisting of methanol:tetrahydrofuran (THF), 1:1 v/v, containing 0.01% BHT. One ml of aliquots was cleared through a 0.20 µm filter prior to HPLC analysis.

Carotenoids were analyzed by a Dionex Ultimate 3000 UHPLC system (Thermo Fisher Scientific) with an Onyx C18 column (100 × 4.6 mm, 5 µm particle size) (Phenomenex), at 35 °C. Twenty µl of the sample was loaded onto the column, and molecules were eluted isocratically with a mixture of acetonitrile:methanol:ethyl acetate 60:30:10 v/v/v containing 0.1% v/v triethylamine (TEA). The flow rate was 1.0 ml min^−1^, and the total run time was 10 min. Lycopene and β-carotene were identified based on their retention time and UV–Vis spectra, as well as by comparison with standard lycopene and β-carotene (Merck). The purified standards were also used to build up calibration curves in the 0.8–51 µg/ml range for lycopene at 472 nm, and in the 0.2–60 µg/ml range for β-carotene at 450 nm.

α-Tocopherol analysis was performed using a Dionex Ultimate 3000 UHPLC system (Thermo Fisher Scientific). Briefly, 2.5 µl of the sample was loaded onto a VisionHT C18 HL column (100 × 2.0 mm, 1.5 µm particle size) (Grace & Co.-Conn., Deerfield, IL, USA), at 40 °C. Molecules were eluted isocratically with 95% methanol. The flow rate was 0.3 ml min^−1^, and the total run time was 7 min. α-Tocopherol was identified based on its retention time and UV spectra, as well as by comparison with the α-tocopherol standard (Merck). The purified standard was also used to establish calibration curves in the 4.0–250 µg/ml range at 292 nm.

### Extraction and analysis of polyphenols

Extraction and analysis of polyphenols from tomato powder were carried out according to Muir and coworkers^55^. Briefly, 0.5 g of tomato powder was dissolved in methanol (5 ml). The suspension was stirred for 30 min, at 25 °C, and centrifuged at 2000 g for 10 min. Five hundred µl of supernatant were added to 500 µl of 0.1% v/v TFA, pH 2.5. After vortexing the mixture, the extract was cleared using a 0.20 µm filter.

Polyphenols were analyzed using a Dionex Ultimate 3000 UHPLC system (Thermo Fisher Scientific) with a Polaris 5 C18-A column (150 × 4.6 mm, 5 µm particle size) (Varian, Mulgrave, AUS), at 30 °C. The injection volume was 20 µl. The elution was carried out with a gradient of solution B (99.9/0.1 v/v acetonitrile/TFA) in solution A (99.9/0.1 v/v water/TFA, pH 2.5), at a flow rate of 1.0 ml min^−1^. Acetonitrile ramped from 0 to 42% in 20 min and remained constant at 42% for 27 min before restoring the initial conditions in 28 min. The phenolic compounds of interest were identified based on their retention time and UV spectra and by comparison with the standards of chlorogenic acid, gallic acid, ferulic acid, and kaempferol (Merck). The purified standards were also used to establish calibration curves in the 2.5–40 µg/ml range for chlorogenic acid at 325 nm, 2.5–38 µg/ml range for gallic acid at 290 nm, 0.2–20 µg/ml range for ferulic acid at 321 nm, and 0.3–10 µg/ml range for kaempferol at 370 nm.

### Germination assay

The effects of triacontanol on seed germination were analyzed on 200 tomato seeds, half soaked in 70 µM triacontanol and half in water for 4 days at 25 °C. Primed seeds were then sowed in a small-scale hydroponic system and transferred to the growth chamber^56^. After one week, the germination rate was determined.

### Hormone extraction and analysis

Extraction of hormones from seeds or leaves of salt-stressed and control plants, which were previously biostimulated or not with triacontanol, was carried out according to Trupiano and colleagues^57^. For HPLC analysis, an LC-20 Prominence HPLC system was used (Shimadzu, Kyoto, JP); it comprised an LC-AT quaternary gradient pump, an SPD–M20A photodiode array detector, and an autosampler SIL-20 AH. The injection volume was set at 20 µl, and the sample separation was performed with a Gemini–NX C18 column (250 × 4.5 mm, 5 µm particle size) (Phenomenex). Hormone elution was carried out with a gradient of solution B (99.9/0.1 v/v acetonitrile/TFA) in solution A (99.9/0.1 v/v water/TFA), running at a flow rate of 1.5 ml/min, at 45 °C. Solution B ramped from 15 to 30% in 5 min, from 30 to 50% in further 5 min, and increased to 80% in 2 min; finally, the gradient returned to the starting conditions in 3 min. The compounds of interest were identified based on their retention time and the corresponding UV spectra as well as by comparison with standard abscisic acid (ABA) (Duchefa Biochemie, Haarlem, NL) and gibberellins (GAs) (Merck). The latter ones were also used to build up calibration curves in the 5–200 µg/ml range, at wavelengths of 254 nm for ABA and 205 nm for GAs.

### Protein extraction, digestion, and peptide fractionation

Proteins from leaves and fruits of triacontanol-stimulated and control tomatoes were extracted through a modified version of the trichloroacetic acid (TCA)-acetone precipitation method, as previously reported^58^. Two independent biological replicates were analyzed for each experimental condition, each containing 20 leaves and 30 fruits, respectively. In particular, the leaves and fruits were homogenized in liquid N_2_, and 1 g of the powder was resuspended in 30 ml of ice-cold acetone supplemented with 10% w/v TCA and 0.07% w/v dithiothreitol (DTT). Samples were incubated overnight, at − 20 °C, and then the proteins were precipitated by centrifugation at 35,000 g for 1 h, at 4 °C. Proteins were resuspended in 30 ml of ice-cold acetone containing 0.07% w/v DTT, incubated for 1 h, at − 20 °C, and precipitated by centrifugation at 35,000 g for 1 h, at 4 °C.

After three washing steps in ice-cold acetone with 0.07% w/v DTT, leaf and fruit protein samples were dissolved in parallel with 5 vol of 8 M urea, 50 mM triethylammonium bicarbonate (TEAB), pH 8.5, and protease inhibitor cocktail (Merck) and incubated at 30 °C, for 1 h^59^. Then, protein samples were ultrasonicated at 50 W twice and centrifuged at 10,000 g for 30 min, at 4 °C. The corresponding protein concentration was determined with the Pierce BCA Protein assay kit™ (Thermo Fisher Scientific), according to the manufacturer’s instructions. For all plant tissue samples, 100 μg of proteins were separately diluted in 100 mM TEAB up to a final volume of 100 µl, reduced with 5 μl of 200 mM tris(2-carboxyethylphosphine), for 60 min, at 55 °C, and then alkylated by adding 5 μl of 375 mM iodoacetamide in the dark, for 30 min, at 25 °C. Proteins were precipitated by adding 6 vol of ice-cold acetone and centrifuging at 8000 g for 10 min, at 4 °C, and then air-dried. Protein samples from leaves and fruits of biostimulated and control plants were separately processed and analyzed depending on the plant tissue. They were digested with a freshly prepared solution of trypsin (ratio enzyme: protein 1:50) solved in 100 mM TEAB^60^. Then, leaf peptide digests were labeled with the TMTsixplex Label Reagent Set (Thermo Fisher Scientific) according to manufacturer’s instructions and the matching: leaf coltrol-1-TMT6-126, leaf coltrol-2-TMT6-127, and leaf triacontanol-1-TMT6-128, leaf triacontanol-2-TMT6-129, for 1 h, at 25 °C. A parallel experiment was performed on fruit peptide digests, which were labeled with the TMTsixplex Label Reagent Set (Thermo Fisher Scientific) according to the matching: fruit coltrol-1-TMT6-126, fruit coltrol-2-TMT6-127, and fruit triacontanol-1-TMT6-128, fruit triacontanol-2-TMT6-129. Then, 8 μl of 5% w/v hydroxylamine was added to each sample and mixed for 15 min to quench the derivatization reaction. Independent, comparative experiments were carried out on tagged peptide mixtures from leaf and fruit samples, which were mixed in equimolar ratios (1:1:1:1) and vacuum-dried under rotation depending on the tissue type. TMT-labelled peptides from leaf or fruit samples were separately suspended in 0.1% TFA and fractionated with the Pierce™ High pH Reversed-Phase Peptide fractionation kit (Thermo Fisher Scientific), following the manufacturer’s instructions. After the fractionation step, eight fractions of TMT-labelled peptides from leaf or fruit samples were separately dried under vacuum and then dissolved in 0.1% formic acid for final mass spectrometry analysis.

### NanoLC-ESI–MS/MS analysis

Independent analyses were performed for leaf and fruit samples. TMT-labelled peptide fractions from the same tissue typology were analyzed in technical triplicate with a nanoLC-ESI-Q-Orbitrap-MS/MS platform consisting of an UltiMate 3000 HPLC RSLC nano system (Dionex, Sunnyvale, CA, USA) coupled to a Q-ExactivePlus mass spectrometer through a Nanoflex ion source (Thermo Fisher Scientific). Peptides were resolved on an Acclaim PepMapTM RSLC C18 column (150 mm × 75 μm ID, 2 μm particles, 100 Å pore size) (Thermo Fisher Scientific) and eluted with a gradient of 80% acetonitrile containing 0.08% formic acid (solvent B) in aqueous 0.1% formic acid (solvent A), at a flow rate of 300 nl/min^61^. Solvent B ramped from 5 to 60% in 125 min, from 60 to 95% in 1 min, and then remained at 95% for additional 8 min before restoring the initial conditions (5%); finally, the column was equilibrated for 20 min before the next run. The mass spectrometer was set in data-dependent mode with a first full scan (*m/z* range 375–1500, nominal resolution of 70,000), followed by MS/MS analysis of the 10 most abundant ions. The spectra were collected in a scan *m/z* range 110–2000, with a normalized collision energy of 32%, an automatic gain control target of 100,000, a maximum ion target of 120 ms, and a resolution of 17,500. A value of 30 s was utilized for dynamic exclusion.

### Bioinformatic analysis for protein identification, quantification, and functional annotation

Independent bioinformatic analyses were performed for leaf and fruit samples. Raw MS data files from the same tissue type were merged for protein identification and relative quantification with the Proteome Discoverer versus 2.1 software (Thermo Fisher Scientific) using the Mascot algorithm v. 2.4.2^62^ for database search^63^. All the analyses were performed with the following criteria: UniProtKB protein database (*S. lycopersicum*, organism ID 4081, 34658 protein count, 01/23) including the most common protein contaminants; carbamidomethylation at Cys and TMT6plex modification at Lys and peptide N-terminus as fixed modifications; oxidation at Met, deamidation at Asn and Gln, pyroglutamate formation at Gln as variable modifications. Peptide mass tolerance was set to ± 10 ppm and fragment mass tolerance to ± 0.02 Da. Proteolytic enzyme and the maximum missed cleavage were set to trypsin and 2, respectively. Protein candidates assigned based on at least two sequenced peptides and an individual Mascot Score ≥ 30 were considered confidently identified. For quantification, ratios of TMT reporter ion intensities in the MS/MS spectra from raw datasets were used to calculate fold changes between samples. The final peptide assignment was always associated with manual spectra visualization and verification. Results were filtered to a false discovery rate (FDR) value of 1%.

Functional categorization of DRPs was obtained using Mercator software for automated sequence annotation^29^, selecting *S. lycopersicum*, SwissProt-UniProtKB plant proteins, KOG clusters, and InterPro scan, with a cut-off value of 80. Then, information on DRPs was integrated with data from scientific literature and assigned to Bevan functional classes^30^.

### Determination of free proline

Free proline content was determined as already described^58^. Concisely, 0.5 g of eight-week-old tomato leaves were homogenized in liquid N_2_, resuspended in 1 ml of 70% v/v ethanol, and centrifuged for 20 min at 13,000 g, at 4 °C. The supernatant (500 µl) was collected and incubated with 1.5 ml of reagent containing 1% w/v ninhydrin, 60% v/v acetic acid, and 20% v/v ethanol, for 20 min, at 95 °C. The absorbance of the samples was measured at 520 nm with a spectrophotometer, and proline concentration was calculated using proline as the standard.

### Total chlorophyll assay

Total chlorophyll content was determined as already described^25^. One hundred mg of leaves from salt-stressed and control plants, which were previously biostimulated or not with triacontanol, were incubated at 65 °C for 90 min in 5 ml of 80% v/v acetone. Samples were then cooled at 25 °C, and the supernatant was collected. Total chlorophyll concentration was determined by measuring the absorbance at 663 and 645 nm.

### Statistical analysis

Each experiment was performed at least three times. Statistical significance was assessed by unpaired Student’s t-test. All values are expressed as means ± S.E.M.

### Supplementary Information

Supplementary Information 1.

Supplementary Information 2.

Supplementary Information 3.

Supplementary Information 4.

## Data Availability

All the data generated or analyzed during this study are available from the corresponding author upon reasonable request.
